# Towards an Online Seizure Advisory System—An Adaptive Seizure Prediction Framework Using Active Learning Heuristics

**DOI:** 10.3390/s18061698

**Published:** 2018-05-24

**Authors:** Vignesh Raja Karuppiah Ramachandran, Huibert J. Alblas, Duc V. Le, Nirvana Meratnia

**Affiliations:** Pervasive Systems Research Group, University of Twente, Drienerlolaan 5, 7522 NB Enschede, The Netherlands; h.j.alblas@student.utwente.nl (H.J.A.); v.d.le@utwente.nl (D.V.L.); n.meratnia@utwente.nl (N.M.)

**Keywords:** implantable body sensor networks, health-care, EEG, epilepsy, signal processing, machine learning, seizure prediction

## Abstract

In the last decade, seizure prediction systems have gained a lot of attention because of their enormous potential to largely improve the quality-of-life of the epileptic patients. The accuracy of the prediction algorithms to detect seizure in real-world applications is largely limited because the brain signals are inherently uncertain and affected by various factors, such as environment, age, drug intake, etc., in addition to the internal artefacts that occur during the process of recording the brain signals. To deal with such ambiguity, researchers transitionally use active learning, which selects the ambiguous data to be annotated by an expert and updates the classification model dynamically. However, selecting the particular data from a pool of large ambiguous datasets to be labelled by an expert is still a challenging problem. In this paper, we propose an active learning-based prediction framework that aims to improve the accuracy of the prediction with a minimum number of labelled data. The core technique of our framework is employing the Bernoulli-Gaussian Mixture model (BGMM) to determine the feature samples that have the most ambiguity to be annotated by an expert. By doing so, our approach facilitates expert intervention as well as increasing medical reliability. We evaluate seven different classifiers in terms of the classification time and memory required. An active learning framework built on top of the best performing classifier is evaluated in terms of required annotation effort to achieve a high level of prediction accuracy. The results show that our approach can achieve the same accuracy as a Support Vector Machine (SVM) classifier using only 20% of the labelled data and also improve the prediction accuracy even under the noisy condition.

## 1. Introduction

According to the World Health Organization (WHO), chronic diseases kill 40 million people each year, contributing to 70% of the global mortality rate [1]. Epilepsy is one of the chronic neurological disorders characterized by the occurrence of sudden and recurrent abnormal neuronal activity in the brain called seizures [2]. The seizures may vary from being undetectable episodes to long episodes of vigorous shaking of the body [3]. The morbidity and mortality of epilepsy are largely associated with the sudden loss of consciousness, fatal injuries caused by unforeseen seizures, and status epilepticus, where a life-threatening seizure lasts for more than five minutes [4].Epilepsy affects 50 million people globally, and 30–40% of this group cannot be treated with any available medicinal therapy [4].

Advanced Implantable Medical Devices (IMD) such as Deep Brain Stimulator (DBS) [5] and Vagus Nerve Stimulator (VNS) [6] have been extensively researched and developed to significantly reduce the seizure frequency for the patients who do not respond to Anti-Epileptic Drugs (AED) [4,7,8]. IMDs deliver electrical impulses through brain-implanted electrodes to a specific target area in the brain in order to reduce the seizure frequency. However, these IMDs deliver chronic therapy rather than acute targeted therapy, and also lack physiological feedback, which limits their efficacy [8]. For the last 80 years, analyzing the electrical signals from the brain, namely ElectroEncephaloGraphy (EEG) and ElectroCorticoGraphy (ECoG), is a well-established method to describe the physiological process of the seizure development and its impact over different parts of the brain [9]. EEG is a non-invasive method of measuring the electrical signals with electrodes that are placed on the surface of the scalp, whereas ECoG is an invasive method with electrodes that are placed directly on the exposed surface of the brain [10]. The advantages of ECoG over EEG are: (i) the Signal-to-Noise Ratio (SNR) of brain signals in ECoG is higher due to the use of electrodes implanted directly on the brain’s surface (unlike EEG, which uses electrodes mounted on the scalp); (ii) collecting ECoG signal is more convenient for the patient than EEG since the patient does not have to be monitored for long periods; (iii) the impact of artifacts caused by physical movements, electrical activities, and other electrical wearable is almost negligible in ECoG signal [10,11]. Nonetheless, these ECoG signals are highly patient-specific and require medical intervention from experts to analyze these signals.

Applying the principles of statistics to automate the analysis of these physiological signals and to predict the onset of seizure has been researched since 1970 [12,13]. In the last two decades, extensive use of machine learning algorithms to detect the onset of seizure based on EEG and ECoG recordings have been reported. Machine learning algorithms such as Artificial Neural Networks (ANN) and Support Vector Machines (SVM) are used to detect seizure events, given the patient-specific EEG data analysis [14,15,16]. The outcome of these algorithms can be effectively used as physiological feedback to the IMDs, which will then expectantly operate in a closed-loop fashion. As a result, the efficacy of the treatment will be improved by delivering intense targeted stimulation [14].

However, due to the inherent uncertainty of the brain signals and because EEG and ECoG vary a lot depending on age, environments, drug intake, etc., it is extremely challenging, if not impossible, to develop a generic seizure prediction framework for all epileptic patients [17]. In addition, in the case of epilepsy, seizure patterns for each patient not only are unique but also vary with time [18]. As a result, a generic machine learning algorithm will not efficiently work for the same patient as well as for large patient groups [19]. It is evident that constant intervention from a medical expert is required for the generic algorithms to work with the same accuracy in a large patient group.

A more common way to approach this problem is to collect continuous EEG and ECoG data, which captures unique seizure patterns and pre-seizure patterns for individual patients. The collected data is then used to train machine learning algorithms and to develop a patient-specific seizure prediction algorithm. However, this approach is not scalable to large patient groups as developing patient-specific machine learning models requires enormous efforts in collecting, labeling, and training the machine learning algorithms. In addition, a seizure event typically occurs for about 1% of a patients lifetime and, for 99% of the time, an epileptic patient is seizure free. This infrequency in seizure events makes it difficult to capture the naturally occurring seizure patterns that are very different from induced seizure patterns [18].

A notable limitation of the existing methods is the complete exclusion of human-expert in the real-time seizure detection systems, since the human expertise is only utilized for labelling the recorded seizure patterns during the pre-development of the patient-specific seizure-detection systems.

**Figure 1:**
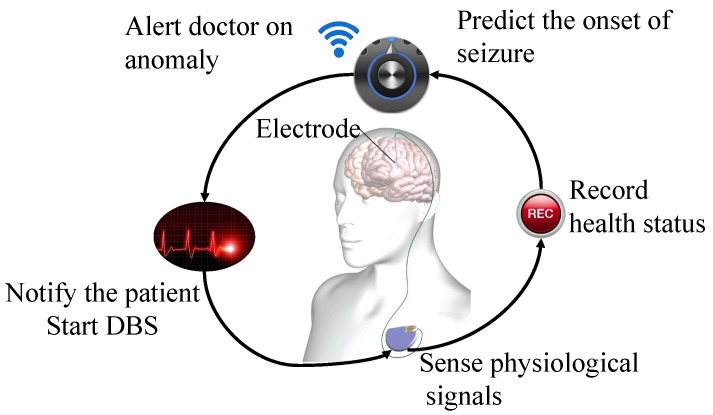
Example of a schematic flow of closed loop operation of the DBS process. Inclusion of the expert is always in loop before any treatment is applied locally. Picture adapted under CC BY 3.0 license from [20].

The first long-term in-man study for a stand-alone seizure advisory system, which predicts the onset of seizures, concluded that substantial disparities are found between reported and detected seizure events, with patient-specific seizure prediction algorithms [19]. (A semi-autonomous operation of advanced medical therapies such as Deep Brain Stimulator (DBS) shown in Figure 1.)

In order to overcome this limitation, authors of [21] used active learning heuristics aiming to reduce the enormous efforts of labeling the data during the training of machine learning model. They developed a scalable and personalized event detection algorithm for infrequent events, like seizure detection in an epileptic patient. Although their results concluded that active learning significantly reduced the number of labels required to train a SVM classifier, the accuracy of detecting an event was not improved.

To this end, in this paper, we propose a lightweight seizure prediction framework that can run on an off-the-shelf ultra-low power hardware, yet can leverage the expert’s knowledge on handling the ambiguous ECoG data in a scalable manner. The main contributions of our paper are:

- Analysis and selection of feature set based on computational time and memory usage,
- Performance analysis of seven different classifiers to select the best performing classifier on a resource constrained platform,
- Application of active learning heuristics and development of a probabilistic seizure prediction framework,
- Performance evaluation of our algorithm based on seizure predictability through simulations.

The rest of this paper is organized as follows. In Section 2, we present the preliminary background for our seizure prediction framework, explaining the nature of the brain signals of an epileptic patient, the main challenges of the online seizure prediction systems, and the related work. In Section 3, we explain the principles of our seizure prediction framework and its active learning heuristics. In Section 4, we explain simulation settings, followed by the validation metrics. In Section 5, we present and discuss the results obtained. Finally, we conclude our work in Section 6.

## 2. Preliminary Background

In this section, we briefly explain the nature of brain signals of an epileptic patient, the main challenges in seizure prediction, and the related work.

### 2.1. ECoGignal

In our study, we use the ECoG signals to design the prediction framework. ECoG is a type of electrophysiological monitoring that measures the electrical activity on specific locations in the brain by implanting a surface-electrode-array through invasive brain surgery [10]. Each electrode in the array is considered as a channel of the measurement. Each channel is sampled at a constant sampling rate producing a constant number of ECoG samples per second. For example, a sampling rate of 400 Hz will produce 400 samples per second on each channel. We will study ECoG of 16 channels.

**Figure 2:**
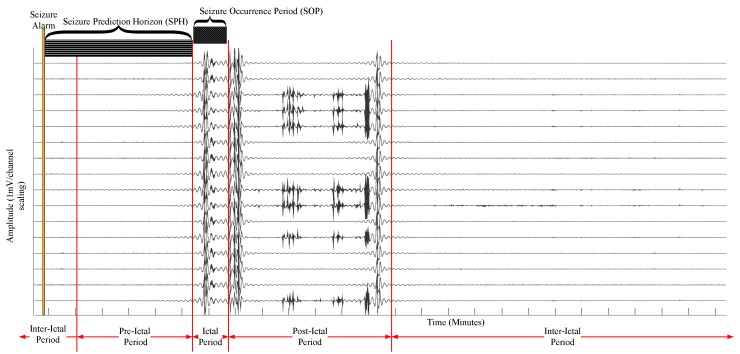
Four states of ElectroCorticoGraph (ECoG) of an epileptic patient [22]. A snapshot of data captured from an array of 16 brain implanted electrodes of an epileptic patient with generalized tonic-clonic seizure. The sampling rate is 500 Hz and is recorded for a total duration of 40 h. Duration of the seizure prediction horizon and the seizure occurrence period are illustrated with respect to the onset of a seizure alarm. (A snapshot of ECoG signal obtained from intracranial ElectroEncephaloGraphy (iEEG) viewer [24].)

As shown in Figure 2, the ECoG is categorized into four periods, namely ictal period, pre-ictal period, post-ictal period, and inter-ictal period [22]. The ictal period is when the actual seizure occurs. The post-ictal is the period shortly after the ictal period when significant spikes in ECoG occur sparsely. The pre-ictal is the period before the seizure occurrence when a patient feels visual auras. The inter-ictal is the seizure-free period between two seizure events [12]. The seizure events are detected in the ictal period. To predict the onset of the ictal period, it is important to detect the pre-ictal period. By doing so, the clinical measures to reduce the impact of seizure events can be taken. Typically, the pre-ictal period can vary from 10 min to 1 h depending on different seizure and patient types; however, there is no straightforward way to define a unique pre-ictal period for future data [23].

By well-established conventions, the Seizure Occurrence Period (SOP) is the time period within which a seizure is expected and the Seizure Prediction Horizon (SPH) is the time period between the onset of prediction alarm and the SOP [19,25,26,27,28]. A seizure prediction framework should analyze real-time ECoG and must generate a time series of binary data classification of pre-ictal denoted by (1) and inter-ictal denoted by (0). These binary classifications can be translated into a seizure warning, and a necessary medical treatment can be provided in real time before the onset of a seizure event. Note that SPH is clinically unknown, but it has to be longer than a pre-ictal period [25]. Ideally, the SPH can be consistently set to the upper bound of the standard pre-ictal period, i.e., 1 h duration, so that SPH will be persistent even for shorter pre-ictal periods say, 5 or 50 min.

### 2.2. Challenges in Seizure Prediction

Although there exist numerous research results in the area of the seizure prediction, most of them are not suitable for real-time detection and execution on low-power devices. It is a well-established convention that EEG data will be collected at a power-deficient sensor node, and the processing of signal will be carried out on a power-surplus base station. Recently, this convention is evidently changing. With the rapid development of the IMDs, the need for seizure prediction algorithm to be able to locally run on such resource constrained environment is paramount. Most of the existing work on seizure prediction arena will neither fit in these resource-constrained hardware platform nor be able to meet the requirements for clinical applications. To this end, we summarize the limitations of seizure prediction concerning the ultra-low power IMDs as follows.

#### 2.2.1. Ultra-Low Power Requirement

As the IMDs are continuously shrinking in size, the need for highly reliable data processing techniques is growing stronger. The physical limitation of resources such as power, and computational capability, is a barrier to run complex algorithms locally on the IMDs. An overview of the technical specification of various IMDs is presented in Table 1. In addition, newly developed algorithms are computationally complex and require more resources to achieve high reliability. An optimum design should intelligently compromise the power consumption and the performance metrics such as reliability and accuracy.

#### 2.2.2. Paradigm Shift from Offline to Online Learning of Seizure Patterns

Neural networks and deep learning have the potential to detect particular seizure related patterns, by extracting and learning features from EEG data without a priori knowledge of the seizure structure. However, the use of these advanced learning algorithms on the IMDs is severely limited due to their large power-hungry computation. With advances in computer architecture such as neuro-morphic computing [34], which mimics the human brain in computations, complex computations with very low power consumption were proven to be possible. Nonetheless, these hardware platforms are still under development to be used for life-critical medical applications. This limits the immediate use of these online learning algorithms on the IMDs.

#### 2.2.3. Uncertainty of Brain Signals

The underlying cause of seizure dynamics in the brain is still not clear. However, the brain has its own regulatory mechanism which could stabilize any abnormal activity in the brain [35]. Because of this, deterministic seizure prediction will not be capable of accurately predicting the onset of seizure, as the seizure event could have been regulated by the brain itself. To address this issue, authors in [18] suggest utilizing a more probabilistic approach such as regression, in which brain signals are continuously filtered through a probability model of seizure events. Moreover, EEG and ECoG are highly uncertain and are susceptible to noises from other parts of the brain that may closely resemble seizure events. The use of other bio-markers such as heart rate and blood pressure has been shown to improve the prediction accuracy [36].

#### 2.2.4. Expert Intervention Is Both Constructive and Destructive

In life-critical seizure prediction systems, where the brain signals are very heterogeneous and uncertain, an expert intervention is needed to overview the outcome of such systems. However, such personalized care with close monitoring of the patient by an expert is unscalable to large size of patient groups. This destructs the purpose for autonomous seizure prediction system, when the outcome of each event has to be monitored by an expert. Nonetheless, when such an autonomous system can detect the ambiguity in the signal with high confidence, an expert can intervene to assist the system with a further course of action. Minimal intervention can be constructive in cases like generic seizure prediction systems.

### 2.3. Related Work

A substantial amount of work has been done in detecting the ictal epochs over the last six decades [37]. It was not until recent years that the focus of research is being shifted to detect the pre-ictal epochs, which is proven to be more helpful in prediction of seizure [19]. A significant number of algorithms have been proposed for seizure prediction at a crowd-sourced seizure forecast competition in Kaggle, set up in the year 2014 by the American Epilepsy Society [37]. A similar approach has been followed by the University of Melbourne together with the American Epilepsy Society in 2016 using a different dataset available in [38]. Most of the top 10 performing algorithms in this competition used SVM based classifiers and used frequency domain features. The description and performance of the top 10 performing algorithms are described in [37].

All of these algorithms are designed to perform well on patient-specific data and with an abundance of labelled data in the pre-ictal class. These algorithms, however, do not focus on the problem of data insufficiency and scalability to large patient groups, but rather focus on the high detection accuracy using patient-specific models. In a practical setting, it is difficult, if not impossible, to collect a large amount of such categorized data in advance for an epileptic patient. A thorough review of existing seizure prediction techniques is presented in [39] by Gadhoumi et al. Other seizure prediction algorithms that aim to be adaptive in their seizure prediction are summarized in Table 2.

One of the major disadvantages of these adaptive seizure prediction algorithms is that they do not have any direct mechanism to handle the ambiguous data that cannot be confidently classified into either pre-ictal class or inter-ictal class. These algorithms aim at changing classifier’s properties such as the distance threshold from the classification plane to improve the classification accuracy. In case of the seizure prediction, in which there is an abundance of ambiguous samples, a simple increase or decrease in the classifier’s threshold will not only decrease the accuracy, but also increase the misclassification rate of the minority class.

The first use of active-learning heuristics to handle the ambiguous data was presented by the authors of [21], where they presented an outlier detector over the results of an SVM classifier and selected the samples that were far away from classification boundary.

In [41], Gupta et al. compared different techniques for active selection and proposed a novel output-based active selection (OAS) method for the active learning framework. In OAS, a meta-classifier called the Bernoulli-Gaussian Mixture Model (BGMM), which combines the base classifier’s uncertainty along with base classifier’s outputs, is generated. The resulting BGMM model has a mixture of distributions consisting of the feature set distribution, crisp label distribution, and soft label distribution. The sub-population of the ambiguous feature set is considered as a hidden variable and identified from the overall population of feature set by estimating the parameters of feature set distribution with respect to crisp and soft label distribution. The set of features with the highest ambiguity is selected using the BGMM.

One of the main advantages of using BGMM over other active selection methods is that it combines the prediction uncertainty with the actual prediction outcome of the classifier to effectively mitigate the querying of morbid samples to be labelled by an expert [41]. The authors applied this algorithm for the task of ensemble classification with an array of re-trainable classifiers using active learning heuristics.

### 2.4. Hypothesis for Our Seizure Prediction Framework

It is a well-established fact that brain signals are inherently uncertain, and pre-ictal signals may vary for different types of seizures [16,39,42]. Moreover, pre-ictal signals can temporally vary for the same seizure and even for the same patient. In this study, we hypothesize that,

Gradual accumulation of pre-ictal signals would improve the overall accuracy of seizure prediction.The inclusion of expert in the system will ensure that pre-ictal signals are labelled correctly in case of ambiguity in classification. However, selecting ambiguous samples to be labelled by an expert without diminishing the autonomous property of the seizure prediction system is a crucial task. By doing so, a seizure prediction system can achieve high prediction accuracy with less number of initial training samples, i.e., with less number of pre-recorded pre-ictal data.

To this end, we aim to develop a seizure prediction framework that is capable of handling the ambiguous samples by seeking feedback from an expert. We will adapt the BGMM to be used for the task of binary classification of pre-ictal and inter-ictal periods from ECoG signals under active learning heuristics. The core of our active learner is based on this BGMM block, which will determine the feature samples that have the most ambiguity. In the following section, we present the seizure prediction framework of which our major contribution is the active learner block and its integration with the machine learning classifier in a closed-loop fashion.

## 3. Adaptive Seizure Prediction Framework

**Figure 3:**
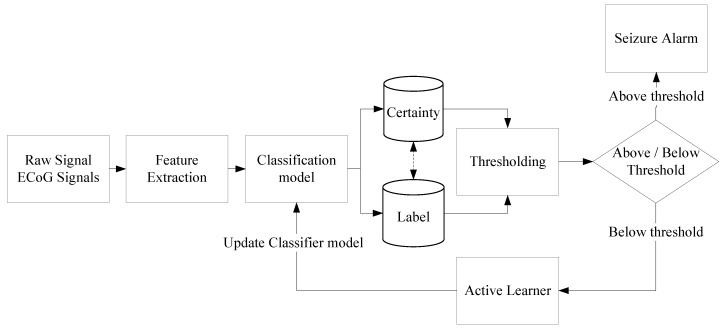
Schematic flow of our seizure prediction framework. The base classifier is expected to output both soft label (or degree of certainty), which is used to determine the certainty of the classification and crisp label (or label) to know the class.

The main aim of our seizure prediction framework is to predict the seizure events with a minimum number of labelled data, and to be scalable for large patient groups. In addition, the complexity of the framework will be kept minimal so that it can be operated on a resource-constrained embedded platforms. As shown in Figure 3, our prediction framework receives the raw ECoG signal over which the set of features will be extracted. The obtained feature set is forwarded to a classification model, which will classify the feature samples into two different classes of labels, i.e., pre-ictal and inter-ictal, with a degree of certainty. During initialization of our framework, the classification model is obtained from minimal labels that are generic to large patient groups. This significantly shortens the initial training periods to obtain a patient-specific classifier model, which will be used in the online phase.

To determine whether a sample is so ambiguous that an expert has to manually annotate, we designed the thresholding block. This block filters the ambiguous samples based on an optimal threshold tuned during the training phase. The threshold is the percentage of the correctly predicted labels accumulated during 1 h. If the outcome is above the threshold, a seizure alarm is triggered. Otherwise, the feature set, the stored labels, and the classification certainty are forwarded to the active learner block, which operates with a core of Bernoulli-Gaussian Mixture Model (BGMM). Labels for the ambiguous samples are obtained from an expert, and the classifier model is updated accordingly. This feedback loop through an active learner would improve the accuracy of the classifier with a minimum number of initial training labels and also the patient-specific accuracy is enhanced over time. In what follows, we elaborate on the functionality of the main building blocks of the framework.

### 3.1. Feature Extraction

The typical approach to analyze the ECoG data using machine learning algorithms is to pre-process the raw signal by computing features. Using features would enhance the classification accuracy and reduce the computational burden. Since the signal conversion from time-domain to frequency-domain consumes a substantial amount of power and considerable processing-time, we focus on the ECoG temporal signals to achieve low-complexity for running on embedded systems. To this end, we selected eight features, which can represent well the pre-ictal epileptiform discharges in the time domain. The features selected are listed below and their definition are stated in [43]:

- “Area—Describes the normalized positive area under the curve.
- Normalized decay—Describes the chance-corrected fraction of signal that is decreasing or increasing.
- Line length—Describes sum of the absolute differences between successive data points.
- Mean energy—Describes mean energy across the data.
- Peak amplitude—Describes the base-10 logarithm of the mean-squared amplitude of the peaks, where a peak is defined as a change from negative to positive in the signal derivative sign.
- Valley amplitude—Describes the base-10 logarithm of the mean-squared amplitude of the valleys, where a valley is defined as a change from positive to negative in the signal derivative sign.
- Normalized peak number—Describes the number of peaks present normalized by the average difference between adjacent data point values.
- Peak variation—Describes the variation between peaks and valleys across both time and values of the data.” [43].

All the above features are computed based on samples collected during non-overlapping sliding windows. For example, a window size of 20 s of ECoG will have 8000 raw samples when sampled at 400 Hz. These time domain features will be extracted from 16 selected channel of the ECoG, which are the most informative channels [44]. As a result for each sliding window, we will have a 128-dimensional vector (16 channels * 8 features), collectively denoted as $set_{t}$ features.

In order to compare the effect of different time-domain and frequency-domain feature sets on classification accuracy, we will also compute the frequency-domain features over the ECoG signals. The frequency-domain set of input features represents the power-in-band properties of the ECoG signals in the commonly studied Berger’s frequency bands, Standard Delta (0–4 Hz), Theta (4–8 Hz), Alpha (8–12 Hz), Beta (12–30 Hz), and Gamma (30–100 Hz). We filter 20 s of ECoG data using a band-pass filter (2nd order Butterworth) with five frequency bands and find the power spectral density by squaring the signal (based on Plancheral theorem to skip the calculation of FFT over ECoG signals). Then, we extract a set of 80 dimensional (16 channels * 5 features) features from non-overlapping 20-s windows of raw ECoG signal, collectively denoted as $set_{f}$ features.

### 3.2. Classification Model

The classifier is an integral part of the prediction framework as shown in Figure 3. We focus on binary classification problem to classify the pre-ictal data and inter-ictal data from patient-specific ECoG. The classification model is generated based on patient-specific ECoG signals. One of the crucial criteria for selecting the classification method is the memory required and the classification time of classifiers. We selected seven classifiers, namely,

- k-Nearest neighbour (kNN) with k = 3,
- k-Nearest neighbour (kNN) with k = 5,
- Support Vector Machine (SVM),
- Logistic regression,
- Naive Bayes,
- Linear Discriminant Analysis (LDA),
- Quadratic Discriminant Analysis (QDA).

We chose three and five neighbors in kNN to generate distinct class boundaries, as a larger k makes boundaries between the classes less distinguishable. The implementation of these classifiers is fairly standard in MATLAB (2017a, MathWorks, Inc., Natick, MA, USA). The classifier is trained with features extracted over 20-s non-overlapping time windows of subject-wise ECoG signals. It is important to note that the entire original dataset will be segmented into 20-s windows and balanced to train the classifier model for different subjects. This process will be explained in detail under Section 4.

### 3.3. Thresholding

The main aim of threshold block in our seizure prediction framework is to temporally analyze the output of the classifier over a period based on a pre-tuned threshold, and to decide if the signal represents the pre-ictal class or the inter-ictal class. For this, we use the segmentation of classifier’s outcome in one hour blocks, i.e., for each 20 s of the raw ECoG signals, the classifier model outputs the class, either pre-ictal or inter-ictal. This outcome is accumulated for 1 h of raw ECoG contributing to 180 classification labels. The threshold is computed based on the ratio between the percentage of the correctly predicted labels accumulated during 1 h. The threshold is computed based on the ratio of the number of correctly predicted labels accumulated during 1 h. If a threshold of 70% is set, it means 126 out of the 180 classification labels should be classified as pre-ictal to trigger a seizure alarm. If it cannot be achieved, the feature set is forwarded to the active learner block together with the soft labels (certainty of the prediction) and crisp labels (prediction of the class) from the classifier. In our experiment, the optimal threshold of 70% was empirically found by varying it using our dataset. As shown in our experimental evaluation, the choice of 70% indeed improved the classification accuracy. In real-world scenarios, this threshold can be changed according to the requirements of the patient and the dataset.

### 3.4. Active Learner

In general, all pre-ictal signals are believed to be unique for each seizure, so it is impossible to capture all the expected pre-ictal signal types in a patient’s lifetime [19]. The re-training process without new labels will not improve the accuracy of the classifier model for unknown pre-ictal signals. An expert’s knowledge is constantly needed to maintain the high accuracy in seizure prediction throughout patient’s lifetime. The advantage of applying the active-learning heuristic to the output of classifier is twofold. One is to improve the prediction accuracy by dynamically updating the classifier model, and the other is to make it scalable by frequently updating the classifier model to achieve patient-non-specific detection of pre-ictal periods. Updating the classifier model requires additional labels and a training phase to derive a model based on the newly obtained labels. In an active learning framework, these additional labels are provided by an expert (otherwise known as oracle w.r.t. active learning terminologies) by observing the ambiguous samples. The process of labeling by the expert and updating the classifier model through training is repeated until a stopping criterion is met. An important aspect of active learning in addressing the issue of expensive labelling over other semi-supervised learning is that active learning explores the instances with the most ambiguity or least confidence, whereas the latter explores the instances with least ambiguity or the most confidence. However, selecting the samples to be labelled by the expert is a crucial task, which decides the success of the active learning framework.

There are various techniques to select the ambiguous samples to be labelled. Examples include the uncertainty-based selection [45], the margin information density criterion [46], and the importance weighting technique [47]. The total number of labelled pre-ictal samples is often lower than the amount of the available inter-ictal samples. Outlier based ambiguous sample selection for active learning will select morbid samplesto be classified in a multi-dimensional feature space, which results in high misclassification. A morbidset of samples is defined as the set of most uncertain samples, which would belong to the same class as predicted by a classifier model, despite the fact that they actually belong to two different classes. Selecting this morbid set of samples to be labelled by an expert will not only increase the labelling cost, but also show no significant improvement in the detection accuracy.

#### 3.4.1. Bernoulli-Gaussian Mixture Model

To prevent morbid samples from being selected to be labelled by an expert, we use BGMM as the active selector block. BGMM will create a mixture of three probability distributions namely, ambiguous-sample and label distributions as Gaussian distributions and their certainty distribution as a Bernoulli distribution. The ambiguity of new samples will be estimated by identifying the probability of the new sample to belong to either pre-ictal or ictal class. The sample with the least probability will be selected to be labeled by an expert. In what follows, we will explain the mathematical modelling of the BGMM block with respect to our problem of ambiguous pre-ictal sample selection from ECoG signals.

Let the feature space defined by $X\subseteq R^{d}$, where *d* denotes the feature dimension and R denotes the measurable set of ECoG signals. Let the binary output space defined by Y⊆Y. We consider the problem of estimating the joint probability density $P_{\mathrm{XY}}$ given a set of input ECoG observations ${\left\{x_{j}\right\}}_{j=1}^{N}$ and output seizure labels ${\left\{y_{j}\right\}}_{j=1}^{L}$, where *L* is the total number of available labels and *N* is the total number of observed ECoG samples. In our case, the labels do not fully represent the observed samples, i.e., 0<L<N, which makes the estimation of $P_{\mathrm{XY}}$ semi-supervised [41]. Hence, the uncertain labels of seizure are defined as ${\left\{y_{j}\right\}}_{j=L+1}^{N}$. Input ECoG observation $x_{j}$ is represented as a marginal 2-component Gaussian mixture distribution, i.e., the inter-ictal epoch and the pre-ictal epoch, and defined as follows:

For c={0,1},

(1) $$\begin{array}{c} \left(x_{j}|y_{j}=c\right)=N\left(x_{j};\mu_{c},\Sigma_{c}\right), \end{array}$$

where $\mu_{c}\;\&\;\Sigma_{c}$ represents the mean and co-variances of the distribution.

The BGMM model also includes the augmented observations ${\left\{u_{j}\right\}}_{j=1}^{L}$ and ${\left\{v_{j}\right\}}_{j=1}^{L}$ where $\left\{u_{j}\right\}\in Y^{K}$ and $\left\{v_{j}\right\}\in {\left\{0,1\right\}}^{K}$, representing the crisp and soft labels (normalized confidence probability) of the classifier’s outcome, respectively. *K* is the maximum number of times an expert can be queried for the label and is set based on the application requirement. The distributions of $\left(u_{j},v_{j}\right)$ are defined as follows. For c={0,1} and m={0,1},

(2) $$\begin{array}{cc} \left(u_{jk}|y_{j}=c\right) & ∼B\left(u_{jk};\pi_{ck}\right), \end{array}$$

(3) $$\begin{array}{cc} \left(v_{jk}|y_{j}=c,v_{jk}=m\right) & ∼N\left(v_{jk};\lambda_{cmk},\sigma_{cmk}^{2}\right), \end{array}$$

where $u_{jk}$ and $v_{jk}$ represent the *k*th component of $u_{j}$ and $v_{j}$, respectively, and B(z;π) denotes a Bernoulli distribution for variable *z* with success probability π.

From these individual distributions, the joint distribution of BGMM is defined as p(x,y,u,v) with parameters $c=\left\{0,1\right\}\;,\;m=\left\{0,1\right\}\;,\;\alpha_{c}$ (mixture proportion), $\Sigma_{c}\in R^{d\times d}\;,\;\mu_{c}\in R^{d}\;,\;\pi_{c}\in \left[0\leq \pi \leq 1\right]\;,\;\lambda_{cmk}\in R^{K\times 2}\;,\;\sigma_{cmk}\in R^{K\times 2}$. The estimation of these parameters is carried out using a an Expectation–Maximization (E–M) algorithm by considering the variables ${\left\{y_{j}\right\}}_{j=L+1}^{N}$ as hidden variables. The cost function is defined as the error in classification due to non-optimal classifier model, i.e.,

(4) $$\epsilon \left(\hat{\theta }\right)=E\left(Y,\hat{\theta }\left(X\right)\right)-E\left(Y,\theta \left(X\right)\right),$$

where E is the expectation operator with respect to the probability measure $P_{XY}$, θ is the optimal classifier model and $\hat{\theta }$ is an estimate of the optimal classifier. The estimation of parameters $\theta =\left(\alpha_{c}\;,\;\mu_{c}\;,\;\Sigma_{c}\;,\;\pi_{c}\right)$ is done by an E-step and an M-step. The E-step creates a function for the expectation of the log-likelihood using the current estimate for the parameters. The M-step computes the parameters by maximizing the expected log-likelihood of the parameters estimated on the E-step, where the E-step is

(5) $$Q\left(\theta ,\theta^{t}\right)=\sum_{j=1}^{N}\sum_{c=0}^{1}\gamma_{j,c}^{\left(t\right)}\left[log\left(\alpha_{c}\right)-\frac{1}{2}log\left|\Sigma_{c}\right|-\frac{1}{2}{\left(x_{j}-\mu_{c}\right)}^{T}\Sigma_{c}^{T}\left(x_{j}-\mu_{c}\right)\right],$$

where in our case, N=L+U, $\gamma_{j,c}^{\left(t\right)}$ is defined by

(6) $$\gamma_{j,c}^{\left(t\right)}=\left\{\begin{array}{cc} I(y_{j}=c), & \mathrm{if}\;1\leq j\leq L,\\ T_{j,c}^{\left(t\right)}, & \mathrm{if}\;(L+1)\leq j\leq N, \end{array}\right.$$

and the $T_{j,c}^{t}$ is given by

(7) $$T_{j,c}^{\left(t\right)}=\frac{N\left({\tilde{x}}_{j};\mu_{c},\sigma_{c}\right)B\left(u_{j};\mu_{c}^{\left(t\right)}\right)\alpha_{c}^{\left(t\right)}}{\sum_{c=0}^{1}N\left({\tilde{x}}_{j};\mu_{c},\sigma_{c}\right)B\left(u_{j};\mu_{c}^{\left(t\right)}\right)\alpha_{c}^{\left(t\right)}}.$$

The M-step is given by

(8) $$\begin{array}{cccc} \pi_{c}^{\left(t+1\right)} & =\frac{\sum_{j=1}^{N}\gamma_{j,c}^{\left(t\right)}u_{j}}{\sum_{j=1}^{N}\gamma_{j,c}^{\left(t\right)}}, & \;\mu_{c}^{\left(t+1\right)} & =\frac{\sum_{j=1}^{N}\gamma_{j,c}^{\left(t\right)}{\tilde{x}}_{j}}{\sum_{j=1}^{N}\gamma_{j,c}^{\left(t\right)}}, \end{array}$$

(9) $$\begin{array}{cccc} \Sigma_{c}^{\left(t+1\right)} & =\frac{\sum_{j=1}^{N}T_{j,c}^{\left(t\right)}\left[{\tilde{x}}_{j}-\mu_{c}^{\left(t+1\right)}\right]\left[{\left({\tilde{x}}_{j}-\mu_{c}^{\left(t+1\right)}\right)}^{T}\right]}{\sum_{j=1}^{N}T_{j,c}^{\left(t\right)}}, & \;\alpha_{c}^{\left(t+1\right)} & =\frac{\sum_{j=1}^{N}\gamma_{j,c}^{\left(t\right)}u_{j}}{\sum_{j=1}^{N}\sum_{c=0}^{1}\gamma_{j,c}^{\left(t\right)}}. \end{array}$$

The output from the BGMM is then forwarded to a *selector* block, which selects the samples with least confidence or the most ambiguity, in order to be labelled by an expert. The least confident sample $\phi_{LC}\left\{x_{j}\right\}$ is selected by $\phi_{LC}\left\{x_{j}\right\}=\left(1-P_{\theta }\left(y_{j}|x_{j}\right)\right)$ [48].

#### 3.4.2. Stopping Criterion

**Figure 4:**
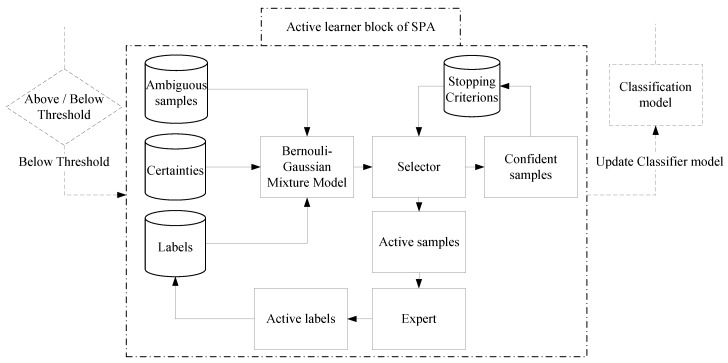
Schematic flow of active learner block based on Bernoulli-Gaussian model. This model takes the ambiguous samples along with the base classifier’s label prediction with its certainty as inputs.

The vital step in active learning is to know when to stop querying the expert; otherwise, it will increase the labelling effort and makes the system unscalable for large patient groups. An active learner must be aware of when to stop the expert querying. For this, we monitor the confidence samples that is observed after the selector block in the active learner (Figure 4). This confidence samples are then used as active feedback to exhibit the confidence in classification accuracy and to stop the active learner from querying the expert.

In order to achieve this, the confidence samples are monitored for each iteration of the active learner block. Querying the expert is stopped when the confidence of the samples remains the same for consecutive iterations of the active learner block. This constant confidence samples directly reflects the accuracy of the base classifier model i.e., the new labels from the expert have improved the classifier model and there are no ambiguous samples to be labelled by an expert. Thus, querying the expert must be stopped immediately.

### 3.5. Seizure Prediction Framework

Our prediction framework is implemented using four main functional blocks, namely,

(i) ExtractFeature$\left(S_{ecog},X\right)$: where the *n* dimensional feature-set (X) is extracted over 20 s time window of raw ECoG signals $(S_{ecog}),$
(ii) Threshold$\left(\hat{Y},PA\right)$: where a prediction alarm PA is set, if 70% of the accumulated labels $\left(\hat{Y}\right)$ are classified as pre-ictal state,
(iii) Classify(X,Y,V,U): where a trained semi-supervised classifier model is used to obtain the set of soft-labels (Y) and crisp-labels (V) , from the input feature-set (X). In addition, the set of uncertain feature samples (U) is obtained based on the soft and crisp-labels of the classifier model,
(iv) Activelearner(X,U,Y,V,A): where a BGMM model based on the feature-set (X), uncertain sample-set (U), soft-label set (Y), and crisp-label set (V) is generated. An E-M algorithm is used to obtain the sample-set (Z) where the confidence of classification of each sample is determined by the probability p(Z). An active sample (A) is selected from the samples of Z, which has the smallest p(Z). The pseudo-code of these individual functional blocks are shown in Algorithm 1.

**Algorithm 1** Pseudo-code of individual functions1: **function**
Extract feature($S_{\mathrm{ecog}},X$)    **input:**
$S_{\mathrm{ecog}}=20\;s;\;\mathrm{all}\;\mathrm{channels}$    **output:**
$X\in R^{K}$ 2:         X = $\left\{x_{1},x_{2},\ldots x_{N}\right\}$ 3: **end function**   4: **function**
Classify(X,Y,V,U)    **input:**
$X\in R^{K}$    **output:**
Y,V,U 5:         **for** each sample in X
**do** 6:                 (Y,V)=θ(X) (acquire soft and crisp labels) 7:         **end for** 8:         **return**
U = $\left\{X\left(P_{Y|V}<80\%\right)\right\}$                                             (obtain ambiguous samples) 9: **end function**10: **function**
Threshold($\hat{Y},PA$) 11:        **if**
$70\%\;\mathrm{of}\;\hat{Y}\;\mathrm{is}\;\mathrm{classified}\;\mathrm{as}\;\mathrm{pre-ictal}\;\mathrm{state}$
**then** 12:                **return**
PA = 1 13:        **else** 14:                **return**
PA = 0 15:        **end if** 16: **end function**
17: **function**
Active learner(X,UY,V,A)      **input:**
X,U,Y,V      **output:**
A 18:        $\theta_{\mathrm{BGMM}}=\mathrm{BGMM}\left(X,U,Y,V\right)$ (Obtain BGMM model) 19:        **for** each sample in U
**do** 20:                $\left(Z,p(Z)\right)=E\_M\left[\theta_{\mathrm{BGMM}}\left(X,Y,V\right)\right]$ 21:        **end for** 22:        $A\;=\;\left(\mathrm{Samples}\;\mathrm{in}\;Z\;\mathrm{with}\;\mathrm{smallest}\;|p(Z)|\right)$ 23: **end function**

As shown in Algorithm 2, our prediction framework sequentially utilizes these functional blocks such that, for every 20 s window of raw ECoG signals, the Extract Feature $\left(S_{ecog},X\right)$ function generates the set of feature vectors. This feature-set is input to the Classify(X,Y,V,U) function, in order to obtain the soft and crisp-labels together with the uncertain feature-subset. Based on these labels, Threshold$\left(\hat{Y},PA\right)$ function triggers a prediction alarm (PA) if more than 70% of the samples are labelled as pre-ictal state. If less than 70% of the labels are classified as pre-ictal, the feature-set together with soft and crisp-labels are forwarded to the active learner. The Activelearner(X,UY,V,A) function then selects the samples with most ambiguity and obtains the label from an expert. The classifier model (θ(X)) is updated based on the newly obtained label and the whole procedure is repeated until the end of the ECoG signal.

**Algorithm 2** Pseudo-code of seizure prediction framework1: **procedure**
Prediction($S_{\mathrm{ecog}}$, PA) 2:         **input:**
$S_{\mathrm{ecog}}$ 3:         **output:**
PA (prediction alarm) 4:         **repeat** 5:                 **for** every 20 s window of $S_{\mathrm{ecog}}$
**do** 6:                      X = Extract feature($S_{\mathrm{ecog}}$) 7:                      (Y,V,U) = Classify(X) 8:                 **end for** 9:                 **while** !(time == 60 mins) **do** 10:                      $\hat{Y}$ = Y (accumulate the labels for 1 h) 11:                 **end while** 12:                 **if** !(Threshold($\hat{Y}$)) **then** 13:                      A = Active Learner($X,U,\hat{Y},V$) 14:                      $Y=\mathrm{expert}\left\{A\right\}$ (Obtain labels) 15:                      update
θ(X)(Update base classifier model) 16:                 **else** 17:                      **return**
PA 18:                 **end if** 19:         **until** End of $S_{\mathrm{ecog}}$ 20: **end procedure**

## 4. Material and Methods

### 4.1. Dataset

The ECoG data used in our work is an open-access data, obtained from the Mayo Systems Electrophysiology Laboratory (MSEL) [49] and intracranial ElectroEncephaloGraphy (iEEG) database [23]. The ECoG data is segmented and labelled as pre-ictal or inter-ictal by human experts. Data is obtained in segments of 10 min sampled at 400 Hz and the total duration is summarized in Table 3.

Epileptic data is obtained from 5 canine and 2 human subjects. The canine epileptic data reflects continuous long-term recordings of dogs with naturally occurring epilepsy. The human patients’ medication was lowered to promote seizures for 7–14 days and the ECoG signals are recorded for a much lower duration in time than the canine subjects. The canine epilepsy is claimed to be an excellent analog for naturally occurring epilepsy in humans [25]. The canine subjects’ dataset contains 16 channels of raw data from implanted electrodes, and human subject Patient 2 has 24 channels of ECoG. Lead seizures are seizures that are recorded without a preceding seizure for more than 7 days for canine subjects and more than 4 h for human subjects. Although inter-ictal data is chosen randomly from a much longer recording, contamination of inter-ictal data by the pre-ictal data is prevented by choosing inter-ictal epochs a week away from the pre-ictal epochs of canine subjects and more than 4 h away from that of human subjects.

#### Imbalanced Dataset and Classification Model

It is important to note that the long hours of data have a very small percentage of pre-ictal segments and a large percentage of inter-ictal segments. As shown in Table 3, we have an imbalance ratio ranging from 20:1 (Dog1 and Dog5) to 2:1 (Patient2) for the inter-ictal and the pre-ictal data. Modelling a classifier on such an imbalanced dataset will result in large classification biases towards the majority class, which is inter-ictal. However, an efficient approach to overcome this problem by modelling a SVM classifier with an unbalanced ECoG dataset [25].

We train the classifier using the balanced dataset, whereas the validation and model selection will be done using the original dataset, which is unbalanced. As we explained earlier, we follow 1 h accumulated windows for seizure prediction based on pre-ictal data. Thus, we split available data into blocks of 1 h and iterate the validation for the number of available pre-ictal blocks, as shown in Table 4. For example, in the Dog5 dataset, we have 5 blocks of pre-ictal data, so the classifier is validated with five-fold cross-validation and iterated 5 times as shown in Table 4. This will ensure that validation is always performed on an out-of-sample dataset, yet each classifier model is validated with its dataset. In addition, the accuracy of the model will be validated based on individual test segments of 1 h rather than extremely short segments, which is clinically relevant [25].

### 4.2. Validation Metrics

The validation process has 3 main goals, which are to evaluate: (i) the performance of the classifiers; (ii) the performance of the active learner, and (iii) the overall seizure prediction accuracy of the framework.

#### 4.2.1. Performance Metrics for the Classifier

The classifier performance is traditionally evaluated using the False Positive Rate (FPR) and False Negative Rate (FNR). Since the goal of the classifier is to classify the pre-ictal class from the inter-ictal class, true positive means correctly classified pre-ictal epoch and true negative means correctly classified inter-ictal epoch.Thus, FPR corresponds to the number of inter-ictal epochs incorrectly classified as pre-ictal epochs, which also results in overall reduction in accuracy of seizure prediction. FNR corresponds to the incorrectly classified pre-ictal epochs as inter-ictal epochs, which also contributes to the overall accuracy, but it is clinically desirable to have very low FNR at the expense of high FPR [25]. The FPR and FNR are calculated over a 1 h time period, in order to match the constraint of prediction horizon and pre-ictal period, i.e., SPH (3–16 h) > pre-ictal period (1 h) (SPH of 3–16 h is based on the range of pre-ictal data available for different patients [refer Table 3]). Nevertheless, we also evaluate performance of the classifiers in terms of the accuracy (A). The accuracy is defined as the ratio of the sum of true positives (correctly classified pre-ictal epoch) and true negatives (correctly classifier inter-ictal epoch) to the sum of real positives (the number of pre-ictal epoch) and real negatives (the number of inter-ictal epoch).

#### 4.2.2. Performance Metrics for the Active Learner

The performance of the active learning process is measured in terms of accuracy (A) of the base classifier, or its complement, error rate (ϵ), with respect to the number of available labels. This is known as the label complexity [41].In general, an ideal active learner will have a label complexity of $O(ln\frac{1}{e})$. In our case, a decline in classification accuracy occurs when the ambiguous feature-samples close to the hyperplane are selected improperly for the active-labelling process.

To evaluate the performance of our active learner with the minimum number of labels, we use a validation method, which is slightly differently from ones of evaluating the performance of the base classifier as shown in Table 11. It is important to note that the base classifier model is not generic for all patients. A personalized training of base classifier model is carried out with each individual subject. Validation of this base classifier model with the active-learning block is carried out still with balanced data, but using different label fractions as shown in Table 11. The expert in our evaluation case is a databaseof labelled feature samples of pre-ictal class, which was not used for training the model. Thus, every time the active learner block is triggered, the database is queried for labelling the unknown samples. In reality, this database will be a trained medical expert. A label fraction is the ratio of the labelled data and the sum of labelled and unlabelled data that is used for validation. In general, if there are *n* pre-ictal epochs available for a subject, a benchmarking of the classifier model is done using *n* pre-ictal epochs, and (n−1) iterations of validation is carried out using sequentially decreasing label fractions. This way, label complexity is measured as a function of classifier accuracy (A).

Misclassification can occur in the base classifier model with real-world data. This misclassification of the base classifier is considered as the noise to the input of active learners. The sensitivity of the active learners in the presence of such random noise in the dataset is measured using noise sensitivity [41]. Noise sensitivity is the measure of error rate ϵ as a function of noise rate η for a fixed label fraction. In the case of subject dog5, we will choose iteration 3 in Table 11 as the validation case, where out of 5 pre-ictal epochs, only 2 pre-ictal epochs are used for training and 3 pre-ictal epochs are used for validation. In general, if there are *n* pre-ictal epoch available for a subject, $\frac{n}{2}-\;1\;\left(\frac{n+1}{2}-\;1\;\mathrm{if}\;n\;\mathrm{is}\;\mathrm{odd}\right)$ is used for training and the noise sensitivity is calculated on the remaining epochs by adding random noise.

#### 4.2.3. Performance Metrics for Seizure Prediction Framework

As mentioned earlier, the seizure alarms are generated after the classification based on the threshold of 70% of the 20 s epochs in 1 h period is classified as the pre-ictal epochs. To compare the performance of our framework with random prediction, we used the Poisson prediction scheme. This Poisson prediction scheme will issue the seizure warning according to an exponentially distributed random time interval with a fixed mean $\lambda_{\mathrm{ran}}$, which is chosen for each subject based on the available dataset according to the time-interval between the lead seizures. For example, in a period of 5 h, if a patient has three events of lead seizure occurring at 1.4, 2.3 and 3.6 h, then $\lambda_{\mathrm{ran}}=1.1$ (averaged inter-seizure duration). With this method, we assume that a priori knowledge of the seizures is randomly compared with our prediction framework. We will use real ECoG data without the balancing process to evaluate the performance of our seizure prediction framework. This will ensure that our classifier model can detect seizures in real unbalanced ECoG data. To evaluate the performance of the prediction framework, we will use the TPR and FPR measures. TPR is defined as the ratio between the total number of correctly predicted seizures and the total number of seizures. The FPR of the prediction framework is defined as the total number of falsely predicted seizure in the total duration of the ECoG signals for each subject. It is important to note that the FPR does not reflect the correctly prediction inter-ictal period, which is true negative. Measuring the TPR and FPR of the framework with the minimum number of labels will emphasize the impact of the active learner in seizure prediction framework.

#### 4.2.4. Platform for Evaluation

All of our algorithms were implemented and validated using existing machine learning libraries in MATLAB2017a installed on a Windows 7 laptop with Intel Core i7 (Santa Clara, CA, USA) 4800MQ @ 2.7GHz CPU and 8 GB RAM. We used the machine learning classifiers with their default implementation and no optimisation of individual classifiers was done, as our work focuses on the relative performance evaluation and not on individual classifiers’ performance. It is important to note that the reported absolute accuracy of the classifier may not be the best possible outcome and can be improved further by optimising the implementation of the classifiers.

## 5. Results and Discussion

In this section, we present and discuss the results.

### 5.1. Feature Selection

**Figure 5:**
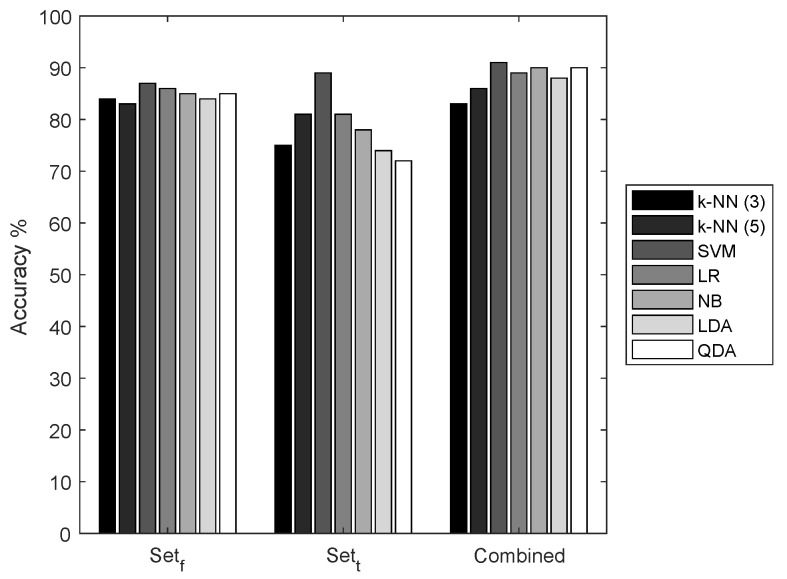
Performance of classifiers for different sets of features.

We selected two sets of features representing the time-domain characteristics and spatial-domain characteristics of the ECoG signals. We compared the performance of seven classifiers with three different feature sets, $set_{t}\;,\;set_{n}$ , and a combination of sets *t* and *f*. It is apparent from the results shown in Figure 5 that the combination of the feature set has the highest accuracy in all the classifiers. However, $set_{t}$ has low performance when compared to the features of $set_{f}$.

For further evaluation of our seizure prediction framework, we intend to use $set_{t}$, mainly for two reasons: firstly, to measure the impact of the active learner on classifier accuracy. With low accuracy, the results of classifiers are not always reliable. With these empirical results, it is known that these features are not completely capable of representing the ECoG signal in higher dimensional feature space, which will result in a large number of ambiguous feature samples close to the classifier’s hyper-plane. Secondly, the calculation of these features is less complex than the other two sets. These features can be implemented in ultra-low power hardware and the power consumption can be greatly reduced when compared to the implementation of other feature sets [50].

#### Feature Extraction on the Dataset

**Figure 6:**
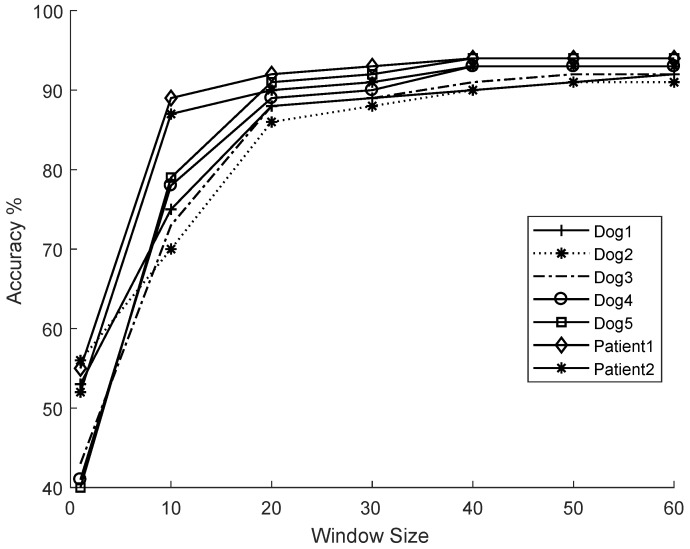
Accuracy (A) of a linear Support Vector Machine classifier as a function of window sizes.

The feature extraction is done on non-overlapping 20 s time windows of raw ECoG signals. Due to the decision of non-overlapping time windows, it is possible that various window sizes will have a significant impact on the classification accuracy (A). We compared the impact of different window sizes on the classification accuracy over different window sizes ranging from 1 s until 100 s in 10 steps. The accuracy in Figure 6 is calculated using a balanced dataset with respect to pre-ictal duration, in a two-fold cross-validation setting.

A SVM classifier with a linear kernel (cost C = 5) is used with features from $set_{t}$ to evaluate the performance of time windows. This accuracy measure is not to reflect the classifier performance, instead to reflect the impact of different choices of window sizes ranging from 1 to 60 s on the classifier performance for different datasets. We found that with our selection of features the classification accuracy of the selected classifiers did not improve after 20 s as shown in Figure 6. Based on these results, we select the 128-dimensional ($set_{t}$) feature vector, which is obtained over a 20 s time window for further evaluations of classifier and active learning framework.

### 5.2. Evaluation of Classifier

In this section, we present and discuss the evaluation and selection of the base classifier model for our seizure prediction framework.

#### 5.2.1. Time and Memory Consumption

We evaluate the performance of seven classifiers with the feature set $set_{t}$ in terms of classification time, i.e., the total time required to classify a 1 h block of raw ECoG signals, using the tic and toc functions of MATLAB. This timing method includes the overhead of the laptop’s background process; however, profiling of all the classifiers is carried out at the same time, with an assurance that the overheads are commonly present during all the timing measurements.

**Figure 7:**
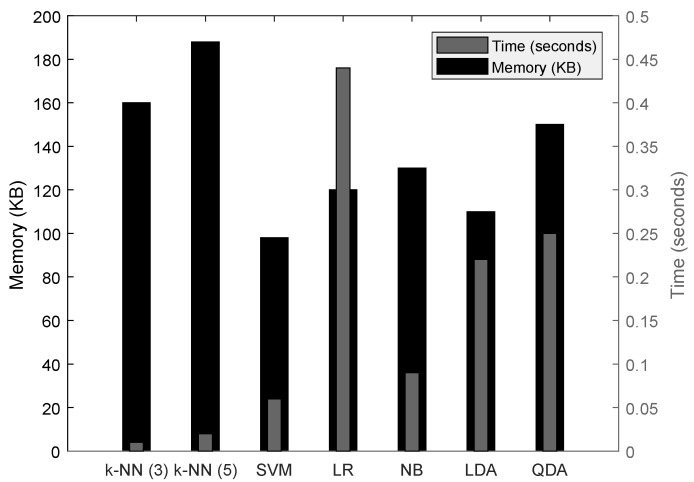
Classification time for 20 s of Electro Cortico Graphy data from 16 channels using $set_{t}$ feature set, timing with Dog5 data.

From Figure 7, it is known that k-NN classifiers require the least time for the classification process because of the minimal computation, but they have the most memory consumption. The most suitable classifier among the list is the SVM, as it requires less memory and requires the least classification time. Based on this result, for further evaluation of the prediction framework, we choose the SVM classifier with $set_{t}$ as the feature set extracted from 20 s of non-overlapped ECoG signals.

In SVM, together with the crisp-label, a degree of certainty, i.e., probability of the output, is also extracted after the classification process using the Platt’s scaling method [51]. In this way, the classifier block outputs a soft-label and a crisp-label after each iteration of the feature set. These labels are forwarded to a threshold block in our prediction framework, which decides whether the classification outcome is eligible for a seizure alarm and can be used for post-processing or if the classifier model needs to be retrained through an active-learning framework.

#### 5.2.2. Evaluation of the Base Classifier Model

In order to evaluate the performance of the classifier with respect to our segmented data, we calculate the performance in each class (pre-ictal and inter-ictal) separately. This ensures that the performance of the classifier is valid for each class. In addition, in this way, we will be able to know how much data from each class is classified as unknown for each subject. For this performance evaluation, we used the segmented data as shown in Table 4. We discuss the results from all iterations for Dog5 dataset in this section.

In Table 12, FPR and FNR are calculated for one hour period to match our assumption of prediction horizon. The feature samples that have a degree of certainty of more than 80% in either class is considered clear samples. These samples can be classified with high confidence into one of the classes. The remaining samples that have a lower degree of certainty are grouped as ambiguous samples. Although a perfect classifier model will have a high degree of certainty and less ambiguous samples near the hyperplane, with this superstitious certainty requirement, we intentionally increase the amount of ambiguous data. This setting will replicate the real-world scenario, where the amount of pre-ictal epochs used for training the classifier will be less than the inter-ictal epochs, naturally increasing the amount of ambiguous data or unknown classes.

From Table 12, it is observed that each iteration using a Dog5 dataset with different validation data has different error rates. In addition, the FPR is always lower than the FNR because of the large amount of inter-ictal data that is used for training the classifier. Iteration5 has the highest number of ambiguous samples, and it is also reflected in the FPR and FNR. The number of ambiguous samples is very high i.e., 78% of the whole validation data used is grouped as ambiguous data. We choose the classifier model, which is developed based on Iteration5 for further evaluation of the active learner block.

In Table 13, we present the performance of the classifier for inter-ictal datasets, with similar metrics. It is evident that the abundance of inter-ictal data has a positive influence on the classifier performance in terms of FPR and FNR, i.e., only a negligent amount of samples are misclassified in the inter-ictal class. In addition, most of the samples are clear and very few samples are ambiguous in all the iterations. It is observed from these results that all classifier models derived from all five of the iterations have quite similar performances for the inter-ictal class. Therefore, we choose the classifier model based on the pre-ictal performance, which is the model Iteration5 for the subject Dog5.

#### 5.2.3. Classifier Model for Each Subjects

A similar process as explained above is used to obtain the base classifier model for each subject. The number of iterations for each subject depends on the number of available one-hour pre-ictal segments. For example, the subject Dog4 has 16 pre-ictal segments so the classifier model has been iterated for 16-fold cross-validation to find the classifier model with the most ambiguous samples as shown in Table 5, Table 6, Table 7, Table 8, Table 9 and Table 10. We present only the iteration that has the most ambiguous samples and its corresponding FPR and FNR are presented in this section. The results of the classifier using datasets of all subjects are presented in Table 14. It is important to note that subjects Patient1 and Patient5 has the highest error rate because of the highest scarcity in pre-ictal data. We selected the classifier model that has the highest amount of ambiguous data in the pre-ictal class.

We also report the performance of the same classifier model with inter-ictal class in Table 15. As explained in the case of Dog5 dataset, the abundance of data in the inter-ictal class has no influence on the selection of classifier model, which is true for all other subjects.

### 5.3. Threshold Value Selection

**Figure 8:**
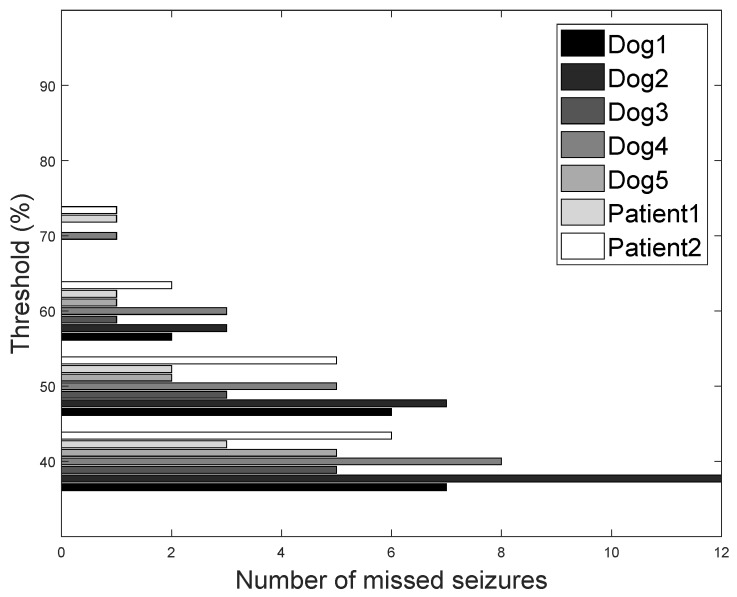
Number of seizure episodes missed from classification as a function of threshold for all dataset.

In order to select the threshold value for our seizure prediction framework, we experimented with different threshold values and measured the number of missed seizure episodes. As mentioned earlier, based on the number of classification labels that are classified as pre-ictal episodes in a 1 h period, a seizure alarm will be triggered. We varied the threshold values from 40% until 90% and calculated how many seizures are missed during the entire period. We present the results in Figure 8, where at a 40% threshold, we have 12 missed seizures for the subjects Dog2, and three missed seizures for Patient1. At 70%, only one seizure is missed for three subjects (Patient2, Patient1, and Dog4) and all the seizures were correctly detected in the other four subjects. At and above 80%, we have correctly detected all the seizures. We will choose a 70% threshold for the rest of the evaluation because the number of accurately predicted seizures is cut off at this threshold value, and it is optimal for improving the classification performance. One may note that step-wise decreasing of the threshold values below 70% results in an increase of missed seizures. Therefore, the use of threshold values lower than 70% is not meaningful.

### 5.4. Evaluation of Active Learners

**Figure 9:**
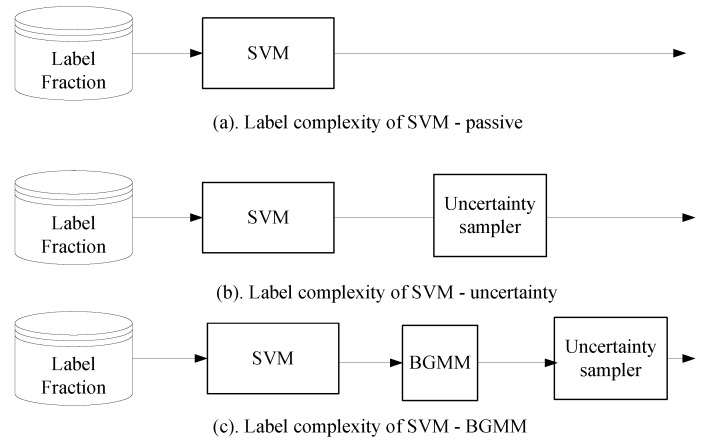
Label complexity of three different settings of the Support Vector Machine classifier. In each setting, the error rate is measured as a function of label fraction.

To compare the performance of the Bernoulli-Gaussian Mixture Model based active learners, we used the Support Vector Machine classifier with semi-supervised setting (or passive setting) and Support Vector Machine with just our uncertainty sampler as shown in Figure 9.

**Figure 10:**
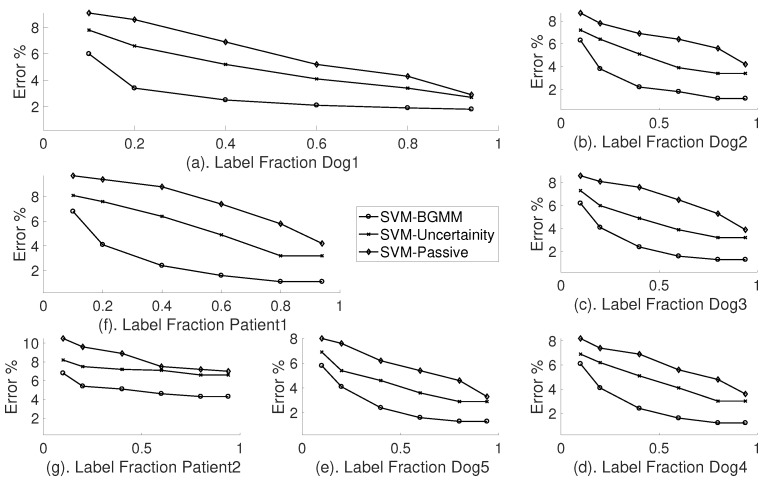
Label complexity of three different settings of the Support Vector Machine classifier. Error rate as a function of label fraction.

The classifier model selected for each subject is validated with different label fractions and the label complexity is presented for each subject separately, as shown in Figure 10. The ratios of different label fractions are derived as shown in Table 11. The model selected from the evaluation of the classifier performance is used to evaluate the active learner block. The error rate is the complement of accuracy A of the classifier. The performance of an ideal active learning in terms of error-rate ϵ should be close to $O\left(\ln\frac{1}{\epsilon }\right)$, whereas, in the semi-supervised case, it is $O\left(\frac{1}{\epsilon }\right)$.

**Figure 11:**
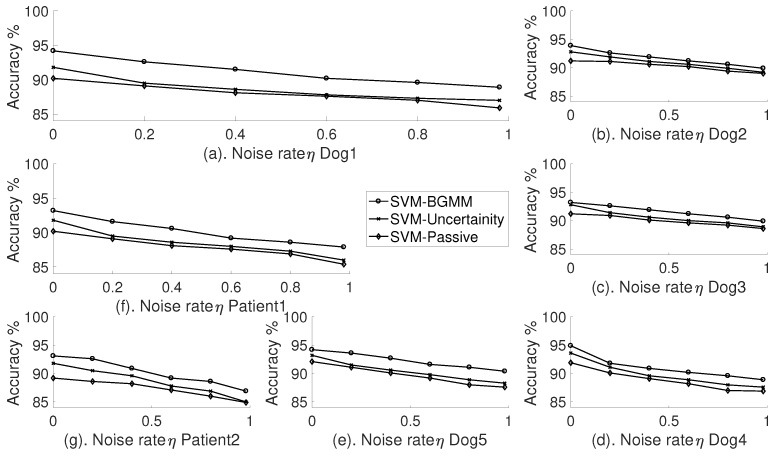
Noise complexity of three different settings of the the Support Vector Machine classifier. Accuracy as a function of noise rate (η).

From Figure 11, it is evident that, for all subjects, the active learner was able to achieve a much lower error rate than the semi-supervised SVM classifier. In Figure 10a, BGMM-based SVM was able to achieve 37% less error rate than semi-supervised SVM, with just 10% of the labels. Although an uncertainty sampler has reduced the error rate by 17% with just 10% of the labels, the complexity is still close to $O\left(\frac{1}{\epsilon }\right)$, which is not desirable. It is evident that the BGMM-based active selection has a clear improvement in the classification error when the amount of labelled data is very low. In addition, for the case of BGMM-based SVM, the error rate saturates at around 1% because of the limitation of the selected classifier model. This complexity is close to $O\left(\ln\frac{1}{\epsilon }\right)$

As explained earlier, the base classifier is prone to misclassification because of the nature of pre-ictal distribution in ECoG signals. We added misclassification noise, i.e., randomly distributed label proportion, to the output of the base classifier. We used the base classifier model that was chosen to have the highest ambiguous sample, and the label fraction was fixed in a way that the number of labelled data segments used for training is always lower than the number of labelled segments used for validation.

In order to evaluate the performance of the active learner block in the presence of such misclassification, we measure the accuracy of the base classifier with three different settings as shown in Figure 11. We measure accuracy of the classifier as a function of different noise proportions η. In the presence of noise, the base classifier in a semi-supervised setting has poor performance. In addition, the uncertainty sampler did not improve the accuracy in the presence of noise. BGMM-based SVM classifiers have significant stability against the misclassification noise. The ability of BGMM to select the active samples based on the estimation probability distribution results in clear isolation of the random noise added to the classification results. It is evident that the misclassification costs can be lowered by the BGMM model; however, choice of classifier model is important to achieve high accuracy. In addition, at very high noise proportion of above 80%, all the settings of the base classifier have similar accuracy in classification.

### 5.5. Evaluation of Seizure Prediction Framework

To compare the seizure prediction performance of our framework, we used the $set_{t}$ features, 20% labelled data with an SVM classifier that has the least memory consumption and shortest execution time. This setting is a valid representation of a low-power, less complex classifier with minimal training phase. In addition, in a practical setting, a seizure prediction framework is expected to work with such a constrained setting. We compare our seizure prediction framework with a Poisson random predictor in terms of True Prediction Rate (TPR) and False Prediction Rate (FPR). The prediction horizon is the time period between a true positive seizure warning and the actual seizure episode.

The performance of the random predictor and our seizure prediction framework is presented in Table 16. The TPR is very low for the Poisson predictor, as it sends the seizure warnings randomly for the seizure occurrence. However, with the minimal prior knowledge, our seizure prediction framework has an average TPR of 87%. In addition, in case of subject Dog3, which has the highest number of seizure events, TPR is very high at 94%.

The prediction horizon of the Poisson predictor is completely random, as the time to raise a seizure warning can be at any random time within inter-ictal periods. This results in very high FPR, which reflects the incorrectly predicted seizure event through the pre-ictal period. In addition, with a random Poisson predictor, the prediction horizons are completely random, reflecting the random seizure events. In our seizure prediction framework, the FPR is very low, reflecting the falsely predicted seizure events, and the prediction horizon is averaged at 21.7 min for all of the subjects.

In order to evaluate our framework for clinical applicability, we calculated the false positives per hour (FP/h). Any seizure prediction to be clinically applicable must not have more than 0.15 FP/h [52]. As shown in Table 17, our seizure prediction tool has very low FP/h for five out of seven subjects. In the case of the Patient dataset, our seizure prediction tool has very high FP/h due to the very low number of seizures occurring over a long duration, i.e., frequency of the recurring seizures is very low. This severely limits the initial training of the model and also reduces the statistical significance between the pre-ictal and inter-ictal data.

### 5.6. Advantages and Limitations of Our Study

In our seizure prediction framework, we enabled an expert-in-the-loop operation, through which an expert can identify the ambiguous pre-ictal signals based on the inter-ictal and ictal episodes. To the best of our knowledge, our study is the first to use active learning heuristics to predict the onset of seizure episodes.We designed our framework to be less complex such that it has very low resource consumption in terms of memory and power. We have used time-domain features for classification, which ensures its implementation in resource-constrained IMD, compared to the frequency domain features. Although we did not present a detailed evaluation of resource consumption analysis of feature extraction and classifier model on an IMD, we relied on extensive literature to support our claim [15,24,43]. Our study also has the lowest false prediction per hour measurements as shown in Table 18 when compared with existing works. For our comparison, we selected the existing works that report the false positives per hour measurement as listed out in [39]. Our results show that on-time prediction of seizures using IMD is plausible, and, through close collaboration with medical experts, the clinical/practical aspects of the approach can be further investigated. Nonetheless, we have indicated clinical/practical aspects such as FP/h and prediction horizon for our seizure prediction framework.

However, our study also has shortcomings. We evaluated our framework based on a small set of intra-cranial measurements. Applicability of our framework to a much broader dataset still needs to be validated. This may result in reiterating the design choices of our framework such as the selection of nonlinear time-domain features for classifier training, an adaptable threshold setting depending on the dataset, and different choices of machine learning classifiers. In the dataset used in this study little to no artefacts was present. Nonetheless, we evaluated the robustness of our framework using noise-complexity, which, in reality, could be caused by artefacts. Naturally occurring artefacts are much more random and might decrease the performance of our framework. The time-domain features are selected based on prior studies that were used for seizure prediction. Although it works perfectly in our dataset, it might not hold true for other datasets. A thorough evaluation with other datasets is needed to overcome these problems.

In this regard, it is important to mention that obtaining the intra-cranial ECoG measurements is not an easy task. An invasive surgical procedure is required to implant the electrode on the surface of the brain. Although more and more pre-recorded intra-cranial datasets are becoming available, limitations still exist in terms of poorly labelled data, lack of information about the method of measurements, and details of the seizure under study. This renders most of the dataset directly unusable for evaluating any seizure prediction system. Moreover, practical application of seizure prediction systems might introduce another set of problems such as the electrode failure, the discrepancy in brain signals, etc. The design choices must be reiterated by clinical evaluation to foresee these practical problems.

## 6. Conclusions

In this work, we presented a low-power, less-complex seizure prediction framework, which was able to achieve almost 95% TPR in seizure prediction with only 20% of the labelled data. An SVM classifier coupled with active learning heuristics is used as the core of the prediction framework. We showed that a scalable expert-in-the-loop operation of life-critical medical devices is possible through our framework. We used openly available intra-cranial ECoG dataset and compared a set of temporal, spatial and a combination of temporal and spatial features to evaluate the performance of seven different classifiers. We selected the best performing classifier with lowest time and memory consumption and used active learning principles to improve the accuracy of the classification with only 20% of the initial labelled data. Finally, a simple threshold-based prediction framework, which leverages the expert knowledge through active learning principles, to improve the classification accuracy is presented and compared with a random seizure predictor. With just 20% of the initial labelled data, we were able to achieve a 95% accuracy in predicting the seizures. The active selection method eliminates the random need of expert knowledge, thereby promising the scalability of our framework. Although we did not evaluate this framework in a clinical setting, we ensured that clinical reprehensibility is achieved through the different choices of data-segmentation, prediction horizon, and feature selection during the development of our seizure prediction framework.

## Figures and Tables

**Table 1:** Technical specifications of implantable medical devices.

Network Parameter	Characteristics of Implantable Medical Devices				
	Pace-Maker [29]	Neural Stimulators [30]	Drug-Delivery Systems [31]	Cochlear Implants [32]	Endoscopy Capsules [33]
Processing duty-cycle	up to 25% of the ON time	up to 50% of the ON time	up to 15% of the ON time	up to 75% of the ON time	up to 100% of the ON time
Processing CPU clock	10–100 kHz	10–100 kHz	10–100 kHz	10–100 MHz	10–100 MHz
Longevity	up to 5 years	up to 5 years	up to 5 years	up to 5 years	up to 2 days
Battery	up to 5 Ah	up to 5 Ah	up to 5 Ah	up to 5 Ah Ah	up to 5 Ah
Memory	up to 128 kB	up to 128 kB	up to 64 kB	up to 2 MB	up to up to 256 MB
Telemetry	Yes	Yes	Yes	Yes	Yes

**Table 2:** A selective summary of seizure prediction methods.

Name of the Work	Machine Learning Method	Dataset Used	Feature Used	Validation Method	Sensitivity	Specificity FP/h
Cook et al. [19]	Decision Tree, k-Nearest Neighbour	Private data	Average energy, Teager-Kaiser energy, line length	Comparison with random predictor based on ground truth	65–100%	Not reported ${}^{1}$
Shiao et al. [25]	Support Vector Machine, intuitive datasegmentation	Kaggle.com, iEEG.org	Band pass filtered power density, Power of Fast Fourier Transform bin. correlation matrix	Ground truth	89–100%	0–0.3 FP/day
Xiao et al. [26]	Adaptive Linear Discriminant Analysis, Adaptive NaiveBayes	Private data	Lyapunov exponent, pairwiseeuclidean distance, T-statistic, Pearson correlation, temporal pattern	Comparison with random predictor based on ground truth	72–82%	0.69–0.93 FP/horizon ${}^{2}$
Parvez et al. [27]	Least square–Support Vector Machine,	Freiburg	Customized phase correlation	Comparison with six existing methods based on ground truth	91–95%	2.4 FP/patient
Aarabi et al. [28]	Rule basedclassification	Freiburg	Lempel-Ziv complexity, Lyapunov exponent, nonlinear interdependence, correlation dimension, correlation entropy, noise-level	Ground truth	86.7–92.9%	0.64–4.69 FP/h
Gadhoumi et al. [40]	Discriminant analysis based classification	Private data	Wavelet energy, entropy, state-similarity using inclusion, persistence, & distance measures	Comparison with random predictor based on ground truth	85–100%	0.1–0.35 FP/h

${}^{1}$ Authors used a different notion to illustrate specificity; ${}^{2}$ Authors calculated false prediction in a specific prediction horizon and not uniformly over an hour.

**Table 3:** Data characteristics used in our study accessed from [49]. Source: [37].

Subject	Sampling Rate in Hz	# of Inter-ictal Segments #(h)	# of Pre-ictal Segments #(h)	# of Lead Seizures	%of Pre-ictal Segments	%of Inter-ictal Segments	Total Duration of Labelled Data (h)
Dog1	400	480 (80 h)	24 (4 h)	8	4.8%	95.2%	84 (hours)
Dog2	400	500 (83)	42 (7)	40	7.8%	92.2%	90
Dog3	400	1440 (240)	72 (12)	18	4.8%	95.2%	252
Dog4	400	804 (134)	97 (16)	27	10.8%	89.2%	150
Dog5	400	450 (75)	30 (5)	8	6.2%	93.8%	80
Patient1	5000	50 (8)	18 (3)	4	26.5%	73.5%	11
Patient2	5000	42 (7)	18 (3)	6	30.0%	70.0%	10

**Table 4:** Separation of 1 h blocks of data for a fair training and validation for Dog1. Each number represents the sequence number of the 1 h blocks [25]. Table 5, Table 6, Table 7, Table 8, Table 9 and Table 10 present the separation of 1 h blocks of data for each subject. However, all iterations are not shown due to their repetitiveness.

Subject	Training Set		Validation Set	
	Inter-Ictal	Pre-Ictal	Inter-Ictal	Pre-Ictal
Dog1 (Iteration1)	2–80	2,3,4	1	1
Dog1 (Iteration2)	1,3–80	1,3,4	2	2
Dog1 (Iteration3)	1,2,4–80	1,2,4	3	3
Dog1 (Iteration4)	1–3,5–80	1,2,3	4	4

**Table 5:** Dog2.

Subject	Tra.–set		Val.–set	
	I . I	P.I	I.I	P.I
Dog2 (It.1)	2–83	2–7	1	1
Dog2 (It.2)	1,3–83	1,3–7	2	2
...					
...					
Dog2 (It.7)	1–6,8–83	1–6	7	7

**Table 6:** Dog3.

Subject	Tra.–set		Val.–set	
	I . I	P.I	I.I	P.I
Dog3 (It.1)	2–240	2–12	1	1
Dog3 (It.2)	1,3–240	1,3–12	2	2
...				
...				
Dog3 (It.12)	1–11,13–240	1–11	12	12

**Table 7:** Dog4.

Subject	Tra.–set		Val.–set	
	I . I	P.I	I.I	P.I
Dog4 (It.1)	2–134	2–16	1	1
Dog4 (It.2)	1,3–134	1,3–16	2	2
...				
...				
Dog4 (It.16)	1–15,17–134	1–15	16	16

**Table 8:** Dog5.

Subject	Tra.–set		Val.–set	
	I . I	P.I	I.I	P.I
Dog5 (It.1)	2–75	2–5	1	1
Dog5 (It.2)	1,3–75	1,3–5	2	2
...				
...				
Dog5 (It.5)	1–4,6–75	1–4	5	5

**Table 9:** Patient1.

Subject	Tra.–set		Val.–set	
	I . I	P.I	I.I	P.I
Patient1 (It.1)	2–8	2,3	1	1
Patient1 (It.2)	1,3–8	1,3	2	2
Patient1 (It.3)	1,2,4–7	1,2	3	3

**Table 10:** Patient2.

Subject	Tra.–set		Val.–set	
	I . I	P.I	I.I	P.I
Patient2 (It.1)	2–7	2,3	1	1
Patient2 (It.2)	1,3–7	1,3	2	2
Patient2 (It.3)	1,2,4–7	1,2	3	3

**Table 11:** Selection of data blocks for validating active learners.

Subject	Training Set		AL-Validation Set	
	Inter-Ictal	Pre-Ictal	Inter-Ictal	Pre-Ictal
Dog5 (benchmark)	1-75	1,2,3,4,5	71,72,73,74,75	1,2,3,4,5
Dog5 (Iteration1)	1-74	1,2,3,4	75	5
Dog5 (Iteration2)	1-73	1,2,3	74,75	4,5
Dog5 (Iteration3)	1-72	1,2	73,74,75	3,4,5
Dog5 (Iteration4)	1-71	1	72,73,74,75	2,3,4,5

**Table 12:** Performance of the Support Vector Machine classifier in terms of False Positive Rate (FPR) and False Negative Rate (FNR) with Dog5 Pre-ictal dataset using $set_{t}$ feature set.

Iteration	Classifier Performance		Feature Samples (%)	
	FPR (%)	FNR (%)	Clear	Ambiguous
Dog5 (Iteration1)	2.30	23.2	52	48
Dog5 (Iteration2)	3.62	20.7	63	37
Dog5 (Iteration3)	2.45	13.8	37	63
Dog5 (Iteration4)	1.54	22.5	46	54
Dog5 (Iteration5)	4.47	31.3	22	78

**Table 13:** Performance of the Support Vector Machine classifier in terms of False Positive Rate (FPR) and False Negative Rate (FNR) with Dog5 Inter-ictal dataset using $set_{t}$ feature set.

Iteration	Classifier Performance		Feature Samples (%)	
	FPR (%)	FNR (%)	Clear	Ambiguous
Dog5 (Iteration1)	0.01	0.12	92	8
Dog5 (Iteration2)	0.41	0.21	93	7
Dog5 (Iteration3)	0.21	0.87	91	9
Dog5 (Iteration4)	0.14	1.01	97	3
Dog5 (Iteration5)	0.37	0.91	96	4

**Table 14:** Performance of the Support Vector Machine classifier in terms of False Positive Rate (FPR) and False Negative Rate (FNR) for all the subject’s pre-ictal dataset using $set_{t}$ feature set. Classifier performances of only the best iterations are shown.

Subject	Classifier Performance		Feature Samples (%)	
	FPR (%)	FNR (%)	Clear	Ambiguous
Dog1	4.15	34.2	29	71
Dog2	3.74	26.7	24	76
Dog3	3.45	25.8	21	79
Dog4	3.88	29.8	26	74
Dog5	4.47	31.3	22	78
Patient1	8.24	38.5	16	84
Patient2	10.47	41.3	12	88

**Table 15:** Performance of the Support Vector Machine classifier in terms of False Positive Rate (FPR) and False Negative Rate (FNR) for all the subject’s inter-ictal dataset using $set_{t}$ feature set. Classifier performances of only the best iterations are shown.

Subject	Classifier Performance		Feature Samples (%)	
	FPR (%)	FNR (%)	Clear	Ambiguous
Dog1	0.19	0.18	94	6
Dog2	0.12	0.23	95	5
Dog3	0.02	0.11	98	2
Dog4	0.25	0.62	97	3
Dog5	0.37	0.91	96	4
Patient1	0.54	1.45	91	9
Patient2	0.47	1.32	90	10

**Table 16:** Performance of the Support Vector Machine classifier in terms of False Positive Rate (FPR) and False Negative Rate (FNR) for the overall dataset using $set_{t}$ feature set.

Subject	Random Poisson Predictor			Active Learning Predictor		
	TPR	FPR	Prediction Horizon	TPR	FPR	Prediction Horizon
Dog1	0.25	0.98	8 (minutes)	0.88	0.12	14.5 (minutes)
Dog2	0.13	0.96	94	0.95	0.08	20.3
Dog3	0.17	0.94	62	0.94	0.11	15.6
Dog4	0.15	0.99	4	0.88	0.03	18.2
Dog5	0.13	0.97	12	0.88	0.13	36.2
Patient1	0.25	0.98	30	0.75	0.30	20.2
Patient2	0.16	0.97	60	0.83	0.16	23.2

**Table 17:** Performance of active learning based seizure prediction framework in terms of True Prediction per hour (TP/h) and False Prediction per hour (FP/h) for the overall dataset using $set_{t}$ feature set.

Subject	Random Poisson Predictor		Active Learning Predictor	
	TP/h	FP/h	TP/h	FP/h
Dog1	0.05	0.95	0.90	0.10
Dog2	0.09	0.91	0.92	0.08
Dog3	0.06	0.94	0.97	0.03
Dog4	0.02	0.98	0.89	0.11
Dog5	0.04	0.96	0.95	0.05
Patient1	0.02	0.98	0.40	0.60
Patient2	0.03	0.97	0.74	0.26

**Table 18:** Comparison of our study with existing works.

Parameters	This Study	Bandarabadi et al. [53]	Li et al. [54]	Williamson et al. [55]	Aarabi and He [56]	Kuhlmann et al. [57]	Gadhoumi et al. [40]
Feature-type	Time-domain	Time-Frequency	Time-domain	Time-domain	Time-domain	Time-domain	Time-Frequency
Database	iEEG	EPILEPSIAE	FSPEEG	FSPEEG ${}^{1}$	FSPEEG	Freiburg	Private
FP/h	0.03–0.60	0.15	0.11–0.15	0.03–0.07	0.11–0.17	0.64–4.69	0.1–0.35
TP/h	40–97%	78.36%	56–72%	86–95%	79.9–90.2%	50–88%	85%
# of Subjects	7	24 ${}^{2}$	21	21	21	6	17

${}^{1}$ FSPEEG has been discontinued to be complemented and replaced by the larger EPILEPSIAE database; ${}^{2}$ 8 of 24 had intra-cranial EEG recordings.

## Abbreviations

The following abbreviations are used in this manuscript:

DBS	Deep Brain Stimulators
WHO	World Health Organization
BSN	Body Sensor Network
IBSN	Implantable Body Sensor Networks
MICS	Medical Implant Communication Service
IMD	Implantable Medical Devices
EEG	ElectroEncephaloGraph
ECG	ElectroCardioGraph
ECoG	ElectroCorticoGraph
AED	Anti-Epileptic Drugs
ANN	Artificial Neural Networks
SVM	Support Vector Machines
SNR	Signal-to-Noise Ratio
SOP	Seizure Occurrence Period
SPH	Seizure Prediction Horizon
SPA	Seizure Prediction Algorithm
SDA	Seizure Detection Algorithm
BGMM	Bernoulli-Gaussian Mixture Model
E-M	Expectation–Maximization
FPR	False Positive Rate
FNR	False Negative Rate

## References

[1] WHO. http://www.who.int/mediacentre/factsheets/fs355/en/.

[2] Fisher R.S., Boas W.v.E., Blume W., Elger C., Genton P., Lee P., Engel J. (2005). Epileptic seizures and epilepsy: Definitions proposed by the International League Against Epilepsy (ILAE) and the International Bureau for Epilepsy (IBE). Epilepsia.

[3] Fisher R.S., Cross J.H., French J.A., Higurashi N., Hirsch E., Jansen F.E., Lagae L., Moshé S.L., Peltola J., Roulet Perez E. (2017). Operational classification of seizure types by the International League Against Epilepsy: Position Paper of the ILAE Commission for Classification and Terminology. Epilepsia.

[4] WHO. http://www.who.int/mediacentre/factsheets/fs999/en/.

[5] Lee K., Roberts D., Hartov A. (2006). Deep Brain Stimulator. U.S. Patent.

[6] Osorio I., Frei M.G. (2002). Vagal Nerve Stimulation Techniques for Treatment of Epileptic Seizures. U.S. Patent.

[7] Cukiert A., Cukiert C.M., Burattini J.A., Mariani P.P., Bezerra D.F. (2017). Seizure outcome after hippocampal deep brain stimulation in patients with refractory temporal lobe epilepsy: A prospective, controlled, randomized, double-blind study. Epilepsia.

[8] Morace R., Di Gennaro G., Quarato P.P., D’Aniello A., Mascia A., Grammaldo L., De Risi M., Sparano A., Di Cola F., De Angelis M. (2017). Vagal Nerve Stimulation for Drug-Resistant Epilepsy: Adverse Events and Outcome in a Series of Patients with Long-Term Follow-Up. Trends in Reconstructive Neurosurgery: Neurorehabilitation, Restoration and Reconstruction.

[9] Walter W.G. (1938). Critical review: The technique and application of electro-encephalography. J. Neurol. Psychiatry.

[10] Penfield W., Jasper H. (1954). Epilepsy and the Functional Anatomy of the Human Brain.

[11] Yoo Y. (2017). On predicting epileptic seizures from intracranial electroencephalography. Biomed. Eng. Lett..

[12] Viglione S., Walsh G. (1975). Proceedings: Epileptic seizure prediction. Electroencephalogr. Clin. Neurophysiol..

[13] Rogowski Z., Gath I., Bental E. (1981). On the prediction of epileptic seizures. Biol. Cybern..

[14] Ramgopal S., Thome-Souza S., Jackson M., Kadish N.E., Fernández I.S., Klehm J., Bosl W., Reinsberger C., Schachter S., Loddenkemper T. (2014). Seizure detection, seizure prediction, and closed-loop warning systems in epilepsy. Epilepsy Behav..

[15] Shoeb A., Edwards H., Connolly J., Bourgeois B., Treves S.T., Guttag J. (2004). Patient-specific seizure onset detection. Epilepsy Behav..

[16] Villar J.R., Vergara P., Menéndez M., de la Cal E., González V.M., Sedano J. (2016). Generalized models for the classification of abnormal movements in daily life and its applicability to epilepsy convulsion recognition. Int. J. Neural Syst..

[17] Andrzejak R.G., Chicharro D., Elger C.E., Mormann F. (2009). Seizure prediction: Any better than chance?. Clin. Neurophys..

[18] Freestone D.R., Karoly P.J., Cook M.J. (2017). A forward-looking review of seizure prediction. Curr. Opin. Neurol..

[19] Cook M.J., O’Brien T.J., Berkovic S.F., Murphy M., Morokoff A., Fabinyi G., D’Souza W., Yerra R., Archer J., Litewka L. (2013). Prediction of seizure likelihood with a long-term, implanted seizure advisory system in patients with drug-resistant epilepsy: A first-in-man study. Lancet Neurol..

[20] Shamir R., Noecker A., McIntyre C. (2014). Deep Brain Stimulation. Front Young Minds.

[21] Balakrishnan G., Syed Z. Scalable personalization of long-term physiological monitoring: Active learning methodologies for epileptic seizure onset detection. Proceedings of the Fifteenth International Conference on Artificial Intelligence and Statistics.

[22] Litt B., Lehnertz K. (2002). Seizure prediction and the preseizure period. Curr. Opin. Neurol..

[23] Bandarabadi M., Rasekhi J., Teixeira C.A., Karami M.R., Dourado A. (2015). On the proper selection of preictal period for seizure prediction. Epilepsy Behav..

[24] IEEG.ORG. http://www.ieeg.org/.

[25] Shiao H.T., Cherkassky V., Lee J., Veber B., Patterson E.E., Brinkmann B.H., Worrell G.A. (2017). SVM-Based System for Prediction of Epileptic Seizures From iEEG Signal. IEEE Trans. Biomed. Eng..

[26] Xiao C., Wang S., Iasemidis L., Wong S., Chaovalitwongse W.A. (2017). An Adaptive Pattern Learning Framework to Personalize Online Seizure Prediction. IEEE Trans. Big Data.

[27] Parvez M.Z., Paul M. (2016). Epileptic seizure prediction by exploiting spatiotemporal relationship of EEG signals using phase correlation. IEEE Trans. Neural Syst. Rehabilit. Eng..

[28] Aarabi A., He B. (2017). Seizure prediction in patients with focal hippocampal epilepsy. Clin. Neurophysiol..

[29] Hansen D.L., Ecker R.M., Solheim P.R. (2014). Pacemaker Event Queue to Control Device Processor Operating Power. U.S. Patent.

[30] Venkatesan L., Bornzin G.A., Bharmi R., Nabutovsky Y., Shah R., Wilson K. (2016). Systems and Methods for Performing Deep Brain Stimulation. U.S. Patent.

[31] Howell F.W., MacLEOD J., Rodbard D. (2014). Method and System to Indicate Glycemic Impacts of Insulin Infusion Pump Commands. U.S. Patent.

[32] Dueck W.F., Pawsey N.C.K., Sibary P.R., Tasche C. (2016). Cochlear Implant Stimulation. U.S. Patent.

[33] Kaku T. (2012). Endoscope System. U.S. Patent.

[34] Modha D.S. http://www.research.ibm.com/articles/brain-chip.shtml.

[35] Buzsaki G. (2006). Rhythms of the Brain.

[36] Moridani M., Farhadi H. (2017). Heart rate variability as a biomarker for epilepsy seizure prediction. Clin. Study.

[37] Brinkmann B.H., Wagenaar J., Abbot D., Adkins P., Bosshard S.C., Chen M., Tieng Q.M., He J., Muñoz-Almaraz F.J., Botella-Rocamora P. (2016). Crowdsourcing reproducible seizure forecasting in human and canine epilepsy. Brain.

[38] Kuhlmann L. https://www.epilepsyecosystem.org/.

[39] Gadhoumi K., Lina J.M., Mormann F., Gotman J. (2016). Seizure prediction for therapeutic devices: A review. J. Neurosci. Methods.

[40] Gadhoumi K., Lina J.M., Gotman J. (2013). Seizure prediction in patients with mesial temporal lobe epilepsy using EEG measures of state similarity. Clin. Neurophysiol..

[41] Gupta M. (2016). Complexity Reduction for Near Real-Time High Dimensional Filtering and Estimation Applied to Biological Signals. Ph.D. Thesis.

[42] Van Esbroeck A., Smith L., Syed Z., Singh S., Karam Z. (2016). Multi-task seizure detection: Addressing intra-patient variation in seizure morphologies. Mach. Learn..

[43] Wulsin D., Gupta J., Mani R., Blanco J., Litt B. (2011). Modeling electroencephalography waveforms with semi-supervised deep belief nets: Fast classification and anomaly measurement. J. Neural Eng..

[44] Carney P.R., Myers S., Geyer J.D. (2011). Seizure prediction: Methods. Epilepsy Behav..

[45] Dasgupta S. (2011). Two faces of active learning. Theor. Comput. Sci..

[46] Settles B., Craven M. An analysis of active learning strategies for sequence labeling tasks. Proceedings of the Conference on Empirical Methods in Natural Language Processing.

[47] Zhao L., Sukthankar G., Sukthankar R. Importance-weighted label prediction for active learning with noisy annotations. Proceedings of the 21st International Conference on Pattern Recognition (ICPR2012).

[48] Culotta A., McCallum A. Confidence estimation for information extraction. Proceedings of the HLT-NAACL 2004: Short Papers.

[49] MSEL-LAB. http://msel.mayo.edu/data.html.

[50] Page A., Oates S.P.T., Mohsenin T. An ultra low power feature extraction and classification system for wearable seizure detection. Proceedings of the 2015 37th Annual International Conference of the IEEE Engineering in Medicine and Biology Society (EMBC).

[51] Platt J.C. (1999). Probabilistic Outputs for Support Vector Machines and Comparisons to Regularized Likelihood Methods. Advances in Large Margin Classifiers.

[52] Winterhalder M., Maiwald T., Voss H., Aschenbrenner-Scheibe R., Timmer J., Schulze-Bonhage A. (2003). The seizure prediction characteristic: A general framework to assess and compare seizure prediction methods. Epilepsy Behav..

[53] Bandarabadi M., Teixeira C.A., Rasekhi J., Dourado A. (2015). Epileptic seizure prediction using relative spectral power features. Clin. Neurophys..

[54] Li S., Zhou W., Yuan Q., Liu Y. (2013). Seizure prediction using spike rate of intracranial EEG. IEEE Trans. Neural Syst. Rehabilit. Eng..

[55] Williamson J., Bliss D., Browne D., Narayanan J. (2012). Seizure prediction using EEG spatiotemporal correlation structure. Epilepsy Behav..

[56] Aarabi A., He B. (2012). A rule-based seizure prediction method for focal neocortical epilepsy. Clin. Neurophysiol..

[57] Kuhlmann L., Freestone D., Lai A., Burkitt A., Fuller K., Grayden D., Seiderer L., Vogrin S., Mareels I., Cook M. (2010). Patient-specific bivariate-synchrony-based seizure prediction for short prediction horizons. Epilepsy Res..

